# Astragaloside IV is a potential natural neuroprotective agent for stroke: a review

**DOI:** 10.3389/fphar.2025.1718700

**Published:** 2026-01-21

**Authors:** Qiao-Li Zhang, Wen-Xiu Qin, Xiu-Juan Li, Yun-Bo Zhang, Ming Li, Jun-Feng Xu, Zhong-Nan Mao

**Affiliations:** 1 Department of Rehabilitation, The Affiliated Hospital of Gansu University of Chinese Medicine, Lanzhou, Gansu, China; 2 College of Acupuncture and Massage, Gansu University of Chinese Medicine, Lanzhou, Gansu, China; 3 Department of Acupuncture and Moxibustion, National Clinical Research Center for Chinese Medicine Acupuncture and Moxibustion, Tianjin, China; 4 Acupuncture Department, The First Teaching Hospital of Tianjin University of Traditional Chinese Medicine, Tianjin, China

**Keywords:** angiogenesis, apoptosis, Astragaloside IV, neuroprotection, oxidative stress, stroke

## Abstract

Stroke poses a severe threat to human health, with limited therapeutic options currently available. Astragaloside IV (AS-IV), a primary bioactive metabolite derived from Astragalus membranaceus, exhibits multifaceted pharmacological effects, including anti-inflammatory, anti-fibrotic, and antioxidative properties. This review systematically examines recent advances in AS-IV research for stroke treatment, detailing its sources, physicochemical characteristics, mechanisms of action, and therapeutic efficacy in both *in vitro* and *in vivo* models. We critically analyze the potential of AS-IV as an adjunctive therapy for stroke, addressing current research hotspots, challenges, and emerging strategies. Notably, AS-IV synergistically enhances neuroprotection when combined with other plant-derived metabolites. This work provides a theoretical foundation for further development of AS-IV in stroke management. In summary, AS-IV demonstrates significant promise as a natural neuroprotective agent worthy of continued exploration for adjuvant stroke therapy.

## Introduction

Stroke remains a leading cause of global morbidity and mortality. According to the World Stroke Organization’s 2024 report, approximately 94 million people live with stroke sequelae, with 12 million new cases and 7 million deaths annually (Feigin et al., 2025). Ischemic stroke (IS, 65.3%), intracerebral hemorrhage (ICH, 28.8%), and subarachnoid hemorrhage (SAH, 5%) constitute the primary pathological subtypes (Parry-Jones et al., 2025; Lv et al., 2024). IS occurs under conditions of embolism, thrombosis, or systemic hypoperfusion, while ICH and SAH result from vascular rupture (Ananth et al., 2023). These pathological processes involve complex molecular mechanisms, including inflammation and oxidative stress (OS) (Shehjar et al., 2023). Modifiable risk factors (such as environmental, behavioral, metabolic and dietary) significantly contribute to lifetime stroke risk (GBD 2021 Stroke Risk Factor Collaborators, 2024).

Beyond mortality, stroke induces debilitating sequelae such as motor deficits, cognitive impairment, aphasia, and dysphagia (Zhou et al., 2023; Suwaryo et al., 2023; Francisco et al., 2021; Yamaoka et al., 2023; Tater and Pandey, 2021; Jones et al., 2020; Sheppard and Sebastian, 2021), which profoundly impact quality of life and incur annual global costs exceeding $891 billion, Feigin et al. (2023) with a disproportionate burden on developing nations (Gao Y. et al., 2024). Stroke treatment typically involves a combination of strategies aimed at maximizing the restoration of patients’ normal function, focusing primarily on restoring cerebral perfusion and mitigating neural injury (Kuriakose and Xiao, 2020). In recent years, substantial progress has been made in the diagnosis and treatment of stroke; effective recanalization therapy for acute stroke can significantly improve clinical prognosis. However, due to time window limitations, thrombolysis and embolectomy are only applicable to a small subset of patients with acute IS (Zhu et al., 2021). The global rate of intravenous thrombolysis is less than 5%, and fewer than 100,000 patients undergo thrombectomy annually (Song et al., 2025). Additionally, while intra-arterial embolectomy devices can serve as an alternative to clinical thrombolysis, they may cause other complications, resulting in significant limitations (Zeng et al., 2023). Therefore, practical solutions to reduce the burden of stroke are urgently needed to save lives and improve global brain health, quality of life and socioeconomic productivity.

Globally, interest in using natural drugs for stroke treatment has grown substantially, especially natural plant-derived metabolites (Tao et al., 2020). These metabolites offer multiple advantages, including maintaining blood-brain barrier (BBB) function, alleviating cerebral edema, regulating energy metabolism, exerting antioxidative, anti-inflammatory, and anti-apoptotic effects, reducing excitatory amino acid toxicity, enhancing neurogenesis, promoting angiogenesis and synaptogenesis, and demonstrating favorable safety and tolerability profiles, all of which indicate their potential for stroke treatment (Li XH. et al., 2022). For instance, neuroprotective drugs derived from natural medicinal metabolites (e.g., ligustrazine, DL-3-n-Butylphthalide, and Ginkgo biloba extract) have shown robust neuroprotective activity in both *in vitro* and *in vivo* models, with a broad basis for clinical application (Zhu et al., 2021). In recent years, modern pharmacological studies have revealed that Astragaloside IV (AS-IV) plays a critical role in the prevention and treatment of nervous system diseases (Yao et al., 2023). However, there remains a lack of reviews summarizing the pathways and targets through which AS-IV exerts its therapeutic effects in stroke. This review synthesizes evidence on the therapeutic potential of AS-IV in stroke, elucidating its molecular targets and signaling pathways.

## Review methodology

The Web of Science Core Collection (WOSCC) database is the most comprehensive and influential scientific literature database in the world, with a complete citation network (Xu Y. et al., 2023; Tian and Chen, 2024). In this study, we searched the WOSCC database for research on the application of AS-IV in stroke using the following retrieval strategy: (TS = “Stroke*” OR “Cerebrovascular accident*” OR “Cerebral stroke*” OR “Cerebrovascular apoplexy” OR “Brain vascular accident*” OR “Cerebrovascular stroke*” OR “Acute stroke*” OR “Acute cerebrovascular accident*” OR “Apoplexy” OR “CVA*”) AND (TS = “Astragaloside*” OR “Astramembrannin I” OR “Cyclosiversioside F”). Only “articles” and “review articles” in English were included, resulting in 98 articles (77 articles, 21 review articles) published between 1 January 2002, and 31 October 2025. Complete records and cited references of these articles were extracted and downloaded in plain text format, with any issues resolved through discussion. The collected data were imported into VOSviewer (v.1.6.18) and CiteSpace (version 6.3.R1) for bibliometric analysis and graphic visualization.

## Sources and characteristics of AS-IV

Astragalus membranaceus (Fisch.) Bge. (Fabaceae), whose medicinal part is the dried cylindrical root with a pale brownish-yellow or light brown surface, is native to China and was first recorded in Shen Nong Ben Cao Jing (Shennong’s Classic of Materia Medica). It exhibits multiple pharmacological benefits, including tonifying qi and uplifting Yang, solidifying the surface, and acting as an antiperspirant, collecting sores, and generating muscles, with no obvious toxicity reported (Yang et al., 2023; Costa et al., 2019). AS-IV is isolated and extracted from the dried roots of Astragalus membranaceus using techniques such as high-performance liquid chromatography, thin-layer chromatography scanning, and fluorescence spectroscopy (Yu et al., 2014). Also known as astraversianin XIV, astrasieversianin XIV, or cyclosiversioside F, AS-IV is a major active metabolite of Astragalus membranaceus and serves as a quality control marker for this botanical drug (Qu et al., 2024).

AS-IV appears as a white crystalline powder and belongs to the lanosterol-type tetracyclic triterpenoid saponins. Its chemical name is 3-O-β-D-xylopyranosyl-6-O-β-D-glucopyranosyl-cycloastragenol, with a molecular formula of C_41_H_68_O_14_, a relative molecular weight of 784.97, and a CAS number of 84687-43-4 (Chen et al., 2021; Tan et al., 2020). AS-IV is soluble in ethanol, methanol, and acetone, and can be extracted via methods including reflux extraction, ultrafiltration, high-speed centrifugation, water extraction, ultrasonic extraction, and alcohol precipitation (Yang Y. et al., 2022). In terms of chemical stability, AS-IV has a melting point range of 284 °C–286 °C, a boiling point of 895.666 °C ± 65.00 °C, extremely low vapor pressure, and a high flash point of 495.481 °C ± 34.28 °C, all of which indicate excellent thermal stability and low volatility (Chen et al., 2025).

AS-IV exhibits multiple pharmacological effects, including anti-inflammatory, anti-fibrotic, and antioxidative activities, which are mediated by the regulation of distinct signaling pathways (Liang et al., 2023). It has been reported that AS-IV plays a protective role in various diseases, including brain injury, central nervous system (CNS), cardiovascular diseases, respiratory diseases, endocrine disorders, immune system diseases, and pathologies of the kidney, liver and cancer (Zhang et al., 2020). Its linear pharmacokinetics profile and favorable safety profile in rats further support its therapeutic potential (Yang Y. et al., 2022).

## Inhibitory effect of AS-IV on stroke pathogenesis

AS-IV exerts a multi-dimensional inhibitory effect on the pathological progression of stroke, making it a key intervention target for improving disease prognosis. This review expounds the pathological process of AS-IV inhibiting stroke from inhibiting neuroinflammation and immune disorder, regulating OS and ferroptosis, inhibiting neuronal apoptosis, and regulating autophagy and mediator-melting functions (Tables 1, 2).

**TABLE 1:** Neuroprotective effect of AS-IV based on *in vitro* studies.

Author, year	Cell line	Optimal dose and duration	Results	Mechanisms/Pathways	References
Chen, 2024	PC12 cells	100 μM for 12 h	Promote cell survival and regulate signal pathway related proteins	P-Src/P-GRK2	Chen et al. (2024a)
Shi, 2023	hCMEC/D3 cells	10 μM for 12 h	Promote cell proliferation and improve the lumen forming ability	PI3K/Akt/mTOR	Shi et al. (2023)
Hao, 2023	SH-SY5Y cells	20 μM for 48 h	Inhibit apoptosis, regulate autophagy and improving cell viability	AMPK/mTOR	Hao et al. (2023)
Du, 2021	PC12 cells	100 μM for 24 h	Enhance cell vitality and inhibit cell apoptosis	CaSR	Du et al. (2021)
Cao, 2020	BMECs	20 μg/mL for 12 h	Promote cell proliferation, improve cell migration and invasion, and inhibit cell apoptosis	PHLPP-1/Akt	Cao et al. (2020)
Sun, 2020	NSCs	20 μM for 3 days	Promote cell proliferation and regulate protein expression	Akt/GSK-3β	Sun et al. (2020a)
Sun, 2020	NSCs	100 nM for 3 days	Promote cell proliferation	Wnt/β-catenin	Sun et al. (2020b)
Zhang, 2019	HT22 cells	100 μM for 24 h	Promote cell survival, reduce injury, inhibit cell apoptosis and regulate autophagy	P62-LC3	Zhang et al. (2019)
Xue, 2019	Primary cortical neurons	25 μM for 27 h	Improve mitochondrial function and inhibition of neuronal death	PKA/CREB	Xue et al. (2019)

P-Src, phospho-Src; P-GRK2, rat phosphorylated G protein-coupled receptor kinase 2; PI3K, phosphatidylinositol 3-kinase; Akt, protein kinase B; mTOR, mammalian target of rapamycin; AMPK, AMP-activated protein kinase; CaSR, calcium-sensing receptor; PHLPP-1, phosphatase pleckstrin homology domain and leucine-rich repeat protein phosphatase 1; GSK-3β, glycogen synthase kinase-3β; Wnt, wingless/integrated; PKA, protein kinase A; CREB, cyclic adenosine monophosphate response element-binding protein.

**TABLE 2:** Interventional effects of AS-IV on different animal models.

Author, year	Animal model	Intervention	Dose and duration	Results	Mechanisms/Pathways	References
Ma, 2025	MCAO/R SD rats	Intraperitoneal injection	20 mg/kg for 7 days	Reduce the volume of cerebral infarction and brain injury, promote mitochondrial autophagy, reduce OS	PINK1/parkin	Ma et al. (2025)
Yu, 2025	MCAO/R SD rats	Intragastric administration	40 mg/kg for 7 days	Improve nerve function, reduce the volume of cerebral infarction and brain injury, inhibit neuronal apoptosis and reduce the level of serum apoptosis-related factors	JNK/Bid	Yu et al. (2025a)
Zhang, 2024	MCAO/R SD rats	Intraperitoneal injection	20 mg/kg (single)	Reduce nerve function defect, reduce cerebral infarction volume and brain injury, and inhibit focal death of nerve cells	Caspase-1	Zhang et al. (2024)
Chen, 2024	MCAO/R C57BL/6J mice	Intraperitoneal injection	20 mg/kg for 25 days	Neuroprotection and apoptosis inhibition, improving OS and calcium overload	P-Src/P-GRK2	Chen et al. (2024a)
Zhang, 2023	MCAO SD rats	Intraperitoneal injection	20 mg/kg (single)	Regulate neuroinflammation and ferroptosis, improve neurological deficit and reduce neuronal death	Nrf2/HO-1	Zhang et al. (2023)
Shi, 2023	MCAO C57BL/6 mice	Intraperitoneal injection	20 mg/kg for 14 days	Improve nerve function, relieve brain injury and promote angiogenesis	PI3K/Akt/mTOR	Shi et al. (2023)
Hao, 2023	MCAO SD rats	Intragastric administration	50 mg/kg for 7 days	Improve nerve function and brain injury, inhibit neuronal apoptosis and regulate autophagy	AMPK/mTOR	Hao et al. (2023)
Liu, 2022	SAH SD rats	Intraperitoneal injection	20 mg/kg (single)	Reduce brain edema and neuronal death	Nrf2/HO-1	Liu et al. (2022b)
Li, 2022	MCAO/R C57BL/6 mice	Intraperitoneal injection	40 mg/kg (twice)	Improve nerve function, reduce the volume of cerebral infarction, inhibit brain infiltration and NK cell activation after ischemia	STAT 3	Li et al. (2022d)
Shi, 2021	MCAO/R SD rats	Intraperitoneal injection	20 mg/kg for 10 days	Improve neurological function and reduce the volume of cerebral infarction	SIRT1/MAPT	Shi et al. (2021)
Du, 2021	MCAO/R SD rats	Intraperitoneal injection	20 mg/kg (single)	Improve nerve function and relieve brain injury	CaSR	Du et al. (2021)
Li, 2020	Acute cerebral infarction SD rats	Intragastric administration	1.08 g/kg for 6 weeks	Improve cognitive function and up-regulate the levels of TGF-β, Smad1, Smad3 and Smad7	TGF-β/Smad	Li et al. (2020a)
Ni, 2020	MCAO SD rats	Intragastric administration	40 mg/kg for 14 days	Reduce the volume of cerebral infarction, improve neurobehavior and promote neurogenesis	BDNF-TrkB	Ni et al. (2020)
Sun, 2020	Cerebral cortex ischemia C57BL/6 mice	Tail vein injection	200 mg/kg for 3 days	Inhibit nerve cell apoptosis, promote neurogenesis, and relieve anxiety	Akt/GSK-3β	Sun et al. (2020a)
Sun, 2020	Cerebral cortex ischemia C57BL/6 mice	Tail vein injection	2 mg/kg for 3 days	Improve cognition, promote neuroplasticity and neurogenesis	Wnt/β-catenin	Sun et al. (2020b)
Zhang, 2019	MCAO/R SD rats	Intraperitoneal injection	20 mg/kg (single)	Improve nerve function and brain injury, inhibit neuronal apoptosis	P62-LC3	Zhang et al. (2019)

PINK1, PTEN-induced kinase 1; JNK, c-Jun N-terminal kinase; Bid, BH3-interacting domain death agonist; P-Src, phospho-Src; P-GRK2, rat phosphorylated G protein-coupled receptor kinase 2; Nrf2, nuclear factor erythroid 2-related factor 2; HO-1, heme oxygenase-1; PI3K, phosphatidylinositol 3-kinase; Akt, protein kinase B; mTOR, mammalian target of rapamycin; AMPK, AMP-activated protein kinase; STAT, 3, transcription 3; SIRT1, sirtuin 1; MAPT, microtubule-associated protein tau; CaSR, calcium-sensing receptor; TGF-β, transforming growth factor-β; BDNF, brain-derived neurotrophic factor; TrkB, tropomyosin related kinase B; GSK-3β, glycogen synthase kinase-3β; Wnt, wingless/integrated.

### Inhibiting neuroinflammation and immune disorder

Neuroinflammation refers to the complex immune response of the CNS to various endogenous or exogenous stimuli (e.g., misfolded proteins, toxins, and pathogens), leading to inflammatory cell infiltration, gliosis, and neuronal loss in brain tissue (Chen et al., 2022). The inflammatory process in stroke involves the activation of multiple cell types, including microglia, astrocytes, endothelial cells, and leukocytes (Alsbrook et al., 2023). Rapid microglial activation is the earliest hallmark of neuroinflammation (Yan et al., 2022). Microglia communicate with neurons through physical contact, multiple receptors, and signaling pathways Cornell et al. (2022) and play a dual role in brain injury and repair following cerebral ischemia-reperfusion injury (CIRI). Under normal physiological conditions, microglia perform homeostatic functions such as parenchymal surveillance, neurotrophic support, pathogen or debris clearance, and maintenance of synaptic homeostasis and neuronal plasticity (Ayata et al., 2023). When CNS homeostasis is disrupted, microglia (the brain’s primary innate immune cells) sense and respond to pathogen-associated molecular patterns (PAMPs), damage-associated molecular patterns (DAMPs), or nematode-associated molecular patterns, respectively (Ayata et al., 2023).

AS-IV can downregulate pro-inflammatory cytokines at sites of inflammation, upregulate anti-inflammatory cytokines (e.g., transforming growth factor-β, TGF-β), and induce the phenotypic shift of macrophages toward the anti-inflammatory M2 subtype (Liu L. et al., 2022). Through the *in vivo* and histological evaluation of MRI, Li MC. et al. (2024) found that AS-IV significantly reduced the infarct volume, alleviated brain microstructure damage, and improved nerve fiber reorganization in a rat model of IS. The study indicated that AS-IV promotes M2 polarization and reduces M1 polarization of microglia. Additionally, AS-IV inhibits the expression of glycolytic rate-limiting enzymes and energy transport proteins. The *in vivo* study of Li et al. (2021) observed that AS-IV significantly ameliorated long-term brain injury, reduced the expression of M1 microglia/macrophage markers, and increased the expression of M2 microglia/macrophage markers in transient middle cerebral artery occlusion (MCAO) rats model 14 days after CIRI. It also upregulated the mRNA and protein expression of peroxisome proliferator-activated receptor γ. These results suggest that AS-IV improves neuroinflammation in stroke by promoting the phenotypic transition of microglia from M1 to M2.

### Regulating OS and ferroptosis

OS is a key molecular mechanisms involved in stroke pathogenesis (Shehjar et al., 2023). OS is defined as an imbalance between the systemic production of free radicals and the cell’s capacity to detoxify these radicals and counteract their destructive effects on proteins, lipids, and DNA (Yoshikawa and You, 2024). While OS plays a role in regulating multiple biological processes, including immune responses, cell proliferation, steroidogenesis, development, aging, thermogenesis, and cognition (Zhang et al., 2022). However, a hyperoxidative state can trigger cellular and biochemical changes such as endothelial dysfunction, vasculitis, arterial remodeling, and BBB damage. These changes may further lead to cerebral reperfusion injury, cerebral blood flow obstruction, ICH, or hemorrhage (Kumar et al., 2023). Free radicals, particularly reactive oxygen species (ROS), are the primary products of OS and can damage brain tissue, representing a critical pathological mechanism of stroke. Antioxidants that scavenge free radicals can limit neuronal damage following stroke (Feng et al., 2023).

Ferroptosis is a unique OS-induced cell death pathway characterized by glutathione depletion and lipid peroxidation (Li et al., 2022b). It is a non-apoptotic form of cell death distinct from autophagy, necrosis, and apoptosis (Li Y. et al., 2024). Accumulating evidence indicates that ferroptosis plays a key role in nervous system diseases such as stroke, traumatic brain injury, and neurodegenerative diseases (Xu W. et al., 2023). Multiple pharmacological or natural metabolites, as well as cell-intrinsic proteins, have been reported to regulate the process and function of ferroptosis (Tang D. et al., 2021).

The main circulating antioxidant systems include enzymatic antioxidants like superoxide dismutase, catalase, paraoxonase-1, and glutathione peroxidase, which can neutralize ROS (Liu et al., 2024a). Non-enzymatic antioxidants (e.g., glutathione, vitamins C and E, and uric acid) play supplementary roles by scavenging free radicals and protecting cellular metabolites from oxidative damage (Liu et al., 2024a). AS-IV has been shown to exert potent antioxidative effects by scavenging free radicals and reducing lipid peroxidation, thereby alleviating oxidative damage (Zhou et al., 2022). A study demonstrated that following IS, AS-IV protected astrocytes against oxygen-glucose deprivation/reperfusion (OGD/R)-induced injury by inhibiting OS and apoptotic pathways (Yang et al., 2021a). In rats with transient MCAO, researchers found that AS-IV administration reduced brain infarct volume, cerebral edema, and neurological deficits, downregulated the expression of inflammatory cytokines (tumor necrosis factor-α, TNF-α; interleukin-1β, IL-1β; IL-6; and nuclear factor-kappa B, NF-κB), increased the levels of solute carrier family 7 member 11 and glutathione peroxidase 4, decreased lipid ROS levels, and prevented neuronal ferroptosis (Zhang et al., 2023). Liu Z. et al. (2022) used a rat SAH model to evaluate the neuroprotective effect and molecular mechanism of AS-IV against SAH-induced early brain injury, finding that AS-IV enhanced antioxidant capacity, inhibited lipid peroxide accumulation, and alleviated ferroptosis after SAH. *In vitro* and *in vivo* experiments by Wang et al. (2023) further verified that AS-IV inhibited ferroptosis, thereby reducing CIRI.

### Inhibition of neuronal apoptosis

In the pathological progression of stroke, ischemia-hypoxia-induced neuronal apoptosis is a key factor contributing to neurological dysfunction and determining stroke-related mortality and disability (Mao et al., 2022). Therefore, neuronal protection has become a primary focus of effectively salvaging brain function defects. Strategies to achieve this goal include enhancing neuronal protection, promoting neuronal repair and regeneration, and directly mediating neuronal survival or death (Zhao et al., 2022). Currently, there is no effective therapy to prevent neuronal cell death (Yue et al., 2025).

AS-IV exerts neuroprotective effects mainly through regulating mitochondrial function and inhibiting neuronal apoptosis, involving multiple synergistic targets. Mitochondrial dysfunction is a critical initiating event in neuronal damage, as mitochondria not only supply energy for neuronal metabolism but also participate in pivotal pathways that maintain brain homeostasis, such as cell death signaling, free radical generation, and lipid synthesis, Falabella et al. (2021) rendering the brain highly susceptible to mitochondrial impairment (Fairley et al., 2022). Hexokinase catalyzes the first rate-limiting step of glucose metabolism, and among the five hexokinase isoforms, hexokinase II (HKII) is the predominant form due to its high glucose affinity and bifunctional catalytic domain (Tian et al., 2025). Inhibition of HKII expression can reduce neuronal apoptosis, Li et al. (2022c) suggesting that HKII may be a primary regulatory target of AS-IV. *In vitro* and *in vivo* studies by Li et al. (2019) demonstrated that AS-IV promotes the binding of HKII to the outer mitochondrial membrane, which stabilizes mitochondrial membrane potential, inhibits the opening of mitochondrial permeability transition pores, and thereby prevents neuronal apoptosis and DNA damage. Yu L. et al. (2025) compared the neuroprotective effects of different drugs in male rats with IS, finding that AS-IV significantly reduced the release of cytochrome C from mitochondria to the cytoplasm, thereby blocking caspase cascade-mediated neuronal apoptosis. Furthermore, Xue et al. (2019) confirmed that in an *in vitro* OGD model of cultured neurons (a cellular model of ischemia-reperfusion injury), AS-IV significantly enhanced the phosphorylation of protein kinase A (PKA) and cyclic adenosine monophosphate response element-binding protein (CREB). This upregulates the expression of mitochondrial protective proteins, thereby alleviating OGD-induced mitochondrial dysfunction.

Collectively, these findings indicate that AS-IV exhibits multi-target pharmacology. Mitochondrial HKII serves as the primary upstream target mediating mitochondrial protection, while the cytochrome C-caspase pathway and PKA/CREB pathway act as downstream synergistic targets, collectively enhancing its neuroprotective effects.

### Regulating autophagy and media-melting function

Autophagy is a conserved lysosomal degradation pathway. During autophagy, autophagosomes encapsulate cellular cargo and fuse with lysosomes, leading to the degradation of their contents via lysosomal hydrolases (Debnath et al., 2023). The autophagic mechanism is involved in intercellular communication, mediates atypical protein secretion processes, regulates tissue-resident stem cells, modulates immune cell function, and maintains tissue barrier integrity (Klionsky et al., 2021). Autophagy plays a dual role in various diseases (Liu S. et al., 2023). Following stroke, autophagy is activated to varying degrees in response to stress, supporting neuronal survival and overall CNS health (Ajoolabady et al., 2021). This is achieved by maintaining neuronal homeostasis, clearing protein aggregates and damaged mitochondria, sustaining energy balance via the recycling of amino acids, fatty acids, and glucose, and alleviating endoplasmic reticulum stress (Ajoolabady et al., 2021). However, prolonged hypoxia, glucose deprivation, or stroke events can drive excessive autophagy, transforming transiently activated, protective autophagy into chronically activated autophagy that leads to severe cell death (Kuang et al., 2020). A study found that in SH-SY5Y cells, AS-IV can protect against CIRI by inhibiting autophagy and mitochondrial-mediated apoptosis (Hao et al., 2023). Another study confirmed that AS-IV could decrease apoptosis (by balancing the expression of Bcl-2 and Bax) and enhance autophagy (by increasing the LC3II/LC3I ratio and reducing P62 expression), thereby exerting neuroprotective effects against CIRI (Zhang et al., 2019).

## Effect of AS-IV on repair and regeneration after stroke

IS may result from the sudden occlusion of cerebral blood supply arteries due to embolism or local thrombus formation (Wegener et al., 2024). Multiple molecular processes, including inflammation, mitochondrial dysfunction, calcium overload, excitotoxicity, acidosis, OS, and programmed cell death, are associated with IS (Ouyang et al., 2024). In contrast, hemorrhagic stroke is caused by intracranial hemorrhage due to hypertension, aneurysms, or other diseases (Gao X. et al., 2024). Stroke activates multiple pathological cascades that converge on inflammation and vascular dysfunction (Kim et al., 2023). Therefore, post-stroke repair and regeneration are particularly critical. AS-IV exhibits multiple biological activities, including stimulating angiogenesis and reducing ischemia-hypoxia injury (Wang F. et al., 2021). This review elaborates on its mechanisms of post-stroke repair and regeneration, focusing on its effects on neurogenesis, synaptic plasticity, angiogenesis, and BBB repair (Tables 1, 2).

### Promotion of neurogenesis and synaptic plasticity

Adult neurogenesis refers to the process by which neural stem cells (NSCs) or neuronal progenitor cells in the adult brain proliferate, migrate, differentiate into new neurons, mature, and ultimately integrate into functional neural circuits under specific conditions (Li et al., 2025). There are several types of NSCs, such as radial glial cells, neuroepithelial cells, interneuron precursors, basal progenitor cells, and radial astrocytes in the subgranular zone (SGZ), and astrocytes in the subventricular zone (SVZ). These cells contribute to the development of specific neuronal phenotypes and the functional integration of neural circuits, including synaptogenesis and neurotransmitter release (Hussain et al., 2024). As the primary metabolites of the brain, neuronal mitochondria primarily provide direct energy supply and regulate synaptic plasticity, glial mitochondria offer metabolic support, promote myelin protection and repair, and participate in immune regulation, while astrocyte mitochondria support the maintenance of NSC niches and synaptic plasticity (Liu et al., 2024b).

Adult neurogenesis is essential for the functional regeneration of forebrain neural circuits and represents a remarkable example of neural plasticity (Brenowitz et al., 2024). Neurogenesis primarily occurs in the SGZ of the hippocampal dentate gyrus (DG) and the SVZ of the lateral ventricle (Culig et al., 2022). SGZ neurogenesis generates new granule neurons for the DG, participating in hippocampus-dependent memory, while V-SVZ neurogenesis produces new interneurons for the olfactory bulb, supporting olfactory function (Lup and o, 2023). Adult neurogenesis is associated with multiple functions, including memory consolidation, flexible learning and updating, reward learning, emotional contextualization, timestamping, spatial contextualization and navigation, behavioral pattern separation, orthogonalization, avoidance of catastrophic interference, detection and pursuit of novelty, regulation of emotional behavior and mood, and forgetting (Arellano and Rakic, 2024). In the context of aging and neurological diseases, the potential for neuronal renewal and regeneration has become a key driver in the field, attracting interest from researchers, funding agencies, scientific journals, and the public.

An *in vivo* study found that AS-IV increases neurogenesis in the hippocampal DG of mice (Huang et al., 2018). Another combined *in vivo* and *in vitro* study demonstrated that AS-IV inhibits inhibit neuronal apoptosis, promotes neurogenesis, and ultimately alleviates anxiety in mice after stroke (Sun et al., 2020a). Further research supports these findings. A study explored the effect of AS-IV on adult mice after IS via *in vivo* and *in vitro* experiments showed that AS-IV promotes hippocampal neurogenesis and improves cognitive deficits (Sun et al., 2020b). A recent *in vitro* study further confirmed the potential of AS-IV to enhance neuronal survival and axonal regeneration (Lin et al., 2025). These results collectively validate the potential of AS-IV to promote neurogenesis and synaptic plasticity.

### Promotion of angiogenesis

Angiogenesis refers to the physiological process of forming new blood vessels from existing vasculature, a critical process in both health and disease. As highly plastic cells, endothelial cells exhibit distinct dynamic responses at different stages of vascular development, enabling the formation of tissue-specific vascular networks with unique patterns and morphologies (Huveneers and Phng, 2024). In both physiological and pathological contexts, endothelial cells respond to various external and internal signals, which induce them to adopt distinct phenotypes and ultimately drive new blood vessel formation (Thijssen, 2021). Proteolytic enzymes, angiogenic growth factors, and their inhibitors collectively regulate endothelial cell migration and proliferation (Ma et al., 2021).

Following stroke, the ischemic penumbra releases angiogenic factors, inducing endothelial cell proliferation and endothelial progenitor cell migration to form new blood vessels (Yang and Torbey, 2020). Angiogenesis increases blood flow to hypoxic and nutrient-deficient tissues, promotes the survival of neurons at risk by providing nutritional supply, and provides pathways for NSC migration to facilitate neurogenesis (Paro et al., 2022). Therefore, angiogenesis may serve as a key therapeutic target for stroke recovery (Yang and Torbey, 2020).

AS-IV can induce the sustained production of small ubiquitin-related modifier-1 in vascular endothelial cells, thereby improving angiogenesis under hypoxic conditions (Wang B. et al., 2021). In a distal MCAO mice model, Shi et al. (2023) found that moderate-dose AS-IV (20 mg/kg) administered for 14 consecutive days significantly improved long-term neurological function recovery, alleviated histological damage, and promoted cerebral blood flow restoration in ischemic mice. Additionally, AS-IV enhanced microvascular density and the coverage of astrocytes and pericytes around microvessels in the peri-infarct cortex. *In vitro* experiments further showed that AS-IV promotes endothelial cell proliferation and vascular formation after OGD. A study by Ou et al. (2023) used MCAO (in rats) and OGD/R (in human umbilical vein endothelial cells) to simulate stroke revealed that AS-IV ameliorates post-infarction brain tissue damage by promoting angiogenesis. Cao et al. (2020) established an *in vitro* CIRI model of OGD using brain microvascular endothelial cells (BMECs) to evaluate the protective effect of AS-IV on BMECs. The results showed that AS-IV alleviates OGD-induced cell loss by increasing cell proliferation and inhibiting apoptosis. These studies further confirm that promoting angiogenesis is a key biological activity of AS-IV (Wang F. et al., 2021).

### Promotion of BBB repair

Another major vascular dysfunction in stroke is the destruction of BBB. The BBB is a highly selective interface between the blood and the brain, playing a crucial role in maintaining the optimal environment for CNS function and homeostasis. The BBB regulates CNS homeostasis by controlling molecular transport between the blood and the CNS, and prevents blood cells, plasma metabolites, and pathogens from entering the brain by forming a tightly regulated neurovascular unit, which includes endothelial cells, peripheral cells and astrocytes. These cells work together to protect the chemical environment of the nervous system and maintain normal brain function (Alahmari, 2021). Without the BBB, the CNS is vulnerable to invasion by toxins, pathogens, immune cells, or ion disorders, leading to neuronal dysfunction and degeneration (Knox et al., 2022). However, while the BBB protects the CNS from blood-borne toxins and pathogens, its existence complicates drug treatment for CNS diseases, as most chemical and biological drugs are blocked from entering the brain, resulting in low therapeutic efficacy and aggravated side effects (Wu et al., 2023).

AS-IV may be a potential neuroprotective agent targeting the BBB (Li H. et al., 2018). A study by Li et al. (2013) found that in a rat model of brain edema after focal CIRI, AS-IV exhibits potential BBB protective effects, significantly reducing brain water content and improved neurological outcomes. *In vivo* and *in vitro* experiments by Hou et al. (2020) showed that AS-IV effectively protects the BBB and reduces infarct volume by inhibiting endoplasmic reticulum stress-mediated endothelial cell apoptosis. They found that CIRI or OGD/R increases the expression of endothelial cell apoptotic proteins (e.g., Bax, Bcl-2, and caspase-3) and endoplasmic reticulum stress-related proteins (e.g., phosphorylated protein kinase RNA-like endoplasmic reticulum kinase; eukaryotic initiation factor 2α; and C/EBP homologous protein), which were attenuated by AS-IV treatment. A study evaluating the BBB penetration of AS-IV reported a penetration value of 0.49 ± 0.03 (Stępnik and Kukula-Koch, 2020).

Another study on AS-IV, through the combination of literature retrieval, computer simulation, and *in vitro* and *in vivo* experiments, aimed to explore the mechanisms by which botanical drug products with low bioavailability treat nervous system diseases. The results showed that this strategy helps identify that AS-IV can improve BBB permeability after metabolic transformation, thereby exerting therapeutic effects (Hu et al., 2023). Given its low BBB penetration, nanoencapsulation strategies should be explored to enhance its delivery efficiency.

## Mechanistic pathways of AS-IV-related neuroprotection

**FIGURE 1:**
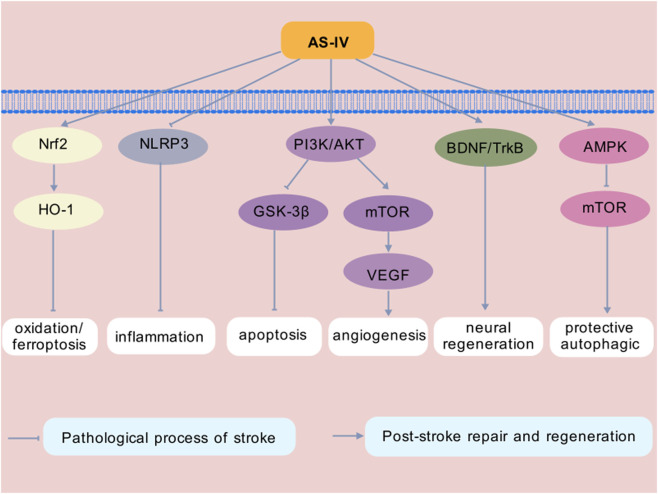
Pathway Related to the Effect of AS-IV for Stroke. Note: Nrf2, nuclear factor erythroid 2-related factor 2; HO-1, heme oxygenase-1; NLRP3, nod-like receptor protein 3; PI3K, phosphatidylinositol 3-kinase; Akt, protein kinase B; GSK-3β, glycogen synthase kinase-3β; mTOR, mammalian target of rapamycin; VEGF, vascular endothelial growth factor; BDNF, brain-derived neurotrophic factor; TrkB, tropomyosin related kinase B; AMPK, AMP-activated protein kinase.

With the continuous advancement of research on the molecular pathological network of stroke, its inherent cellular signaling mechanisms have been gradually and systematically elucidated. Notably, the neuroprotective effects of AS-IV are mediated by multi-pathway regulation. This section discusses the primary signaling pathways involved in AS-IV-mediated neuroprotection, including key pathways such as Nrf2, nod-like receptor protein 3 (NLRP3), PI3K/Akt, BDNF-TrkB, and AMPK/mTOR (Figure 1). The multi-target advantages of AS-IV in neuroprotection provide a critical theoretical foundation for the development of precision treatment strategies and novel drugs based on signaling pathways.

### Nrf2

The activation of Nrf2 pathway can promote the recovery of neurological function after stroke (Duan et al., 2022). This is because Nrf2 regulates most downstream factors and exerts multiple effects, including anti-apoptosis, anti-inflammatory injury, reducing calcium overload and antioxidation, and helps the body maintain redox balance in brain tissue and brain cells (Wang L. et al., 2022). Different natural metabolites can induce Nrf2 to promote health benefits (Gugliandolo et al., 2020). It is found that AS-IV may protect the integrity of BBB in mice by activating the Nrf2 signaling pathway (Li H. et al., 2018). AS-IV can inhibit the expression of the Bax/Bcl-2 ratio by suppressing the C-X-C motif chemokine receptor 4 and downregulating the activation of the phospho-JNK/JNK pathway, ultimately upregulating Nrf2/Kelch-like ECH-associated protein 1 (Keap1) signaling to protect OGD/R-induced astrocytes (Yang et al., 2021a). Additionally, AS-IV can increase the levels of P62 and Nrf2, decrease Keap1 levels, and inhibit ferroptosis by activating the P62/Keap1/Nrf2 pathway, thereby alleviating CIRI (Wang et al., 2023). A study found that AS-IV administration regulates neuroinflammation and neuronal death via the Nrf2/HO-1 signaling pathway, thereby improving delayed ischemic neurological deficits and reducing neuronal death (Zhang et al., 2023). Another study demonstrated that AS-IV inhibits ferroptosis in SAH by activating the Nrf2/HO-1 pathway (Liu Z. et al., 2022). This suggests that activating Nrf2/HO-1 signaling may be a potential target for inhibiting ferroptosis (Yang et al., 2021b). This provides a novel strategy for multi-target stroke treatment.

### NLRP3

NLRP3 inflammasomes have emerged as key mediators of pathological inflammation in many diseases and represent promising therapeutic targets (Coll et al., 2022). NLRP3 inflammasomes are widely expressed in the CNS, where they are activated by PAMPs and DAMPs, leading to cellular microenvironment imbalance. Activated NLRP3 inflammasomes can activate pro-inflammatory caspase-1, triggering secondary inflammatory responses and ultimately causing neuronal damage (K et al., 2024). In recent years, interest in developing drugs that regulate NLRP3 inflammasome activity for the treatment of human diseases has grown rapidly, and several drugs have shown efficacy in animal models and clinical trials (Ma, 2023). An experimental study found that AS-IV may inhibit NLRP3 activation by targeting cell membrane receptors, thereby improving the symptoms of ischemic cerebrovascular diseases (Li Y. et al., 2018). Another study showed that AS-IV inhibits NLRP3-mediated cell death by promoting the expression of Kruppel-like factor 2, alleviating inflammatory damage after cerebral hemorrhage in mice (Wu et al., 2024).

### PI3K/Akt

Increasing evidence confirms that PI3K/Akt signaling pathway plays an important role in regulating cellular life activities and is involved in multiple physiological processes, including cell growth, differentiation, survival, and apoptosis (Yang F. et al., 2022; Wang PC. et al., 2022). In various organs, repair processes are primarily mediated by the PI3K/Akt pathway, especially the CNS (Gu et al., 2022). The PI3K/Akt signal plays an important role in the pathogenesis of IS, Liu et al. (2025) as this pathway can regulate multiple upstream molecules, such as growth factor receptor, G protein-coupled receptor, receptor tyrosine kinase, extracellular signal-regulated kinase, and cytokine (Iranpanah et al., 2023). A study found that AS-IV regulates the PI3K/Akt signaling pathway and promotes CD36 phagocytic function, thereby contributing to hematoma absorption and neurological function prognosis after ICH (Zheng et al., 2022). Another study demonstrated that AS-IV may exert neuroprotective effects on CIRI rats through the PI3K/PKB/Akt signaling pathway (Ding et al., 2025). The PI3K/Akt/mTOR signaling pathway plays a crucial role in promoting neuroprotection and angiogenesis (Wang et al., 2025). A study by Shi et al. revealed that AS-IV promotes angiogenesis in IS by activating the PI3K/Akt/mTOR signaling pathway to increase the expression of vascular endothelial growth factor (VEGF) (Shi et al., 2023). Additionally, glycogen synthase kinase-3β (GSK-3β) is a key intracellular signaling pathway involved in cell apoptosis, survival and proliferation (Wei et al., 2019). A study by Sun et al. found that AS-IV downregulates IL-17 protein both *in vivo* and *in vitro*, exerts antagonistic effects on neurogenesis by regulating the Akt/GSK-3β pathway, and exhibits significant regulatory effects on cell apoptosis (Sun et al., 2020a).

### BDNF-TrkB

BDNF is the most abundant and widely distributed neurotrophic protein in the brain, playing a key role in the development and maintenance of the CNS (Giesler et al., 2024). BDNF interacts with TrkB and subsequently activates the PI3K/Akt and MAPK/extracellular signal-regulated kinase signaling pathways, exerting multiple effects including pro-survival activity, anti-apoptosis, anti-inflammation, and enhancement of dendritic growth and branching (Li C. et al., 2020). Evidence also suggests that BDNF/TrkB signaling is involved in adult hippocampal neurogenesis, with distinct roles in the DG and SVZ (Colucci-D’Amato et al., 2020). An animal experimental study found that AS-IV reduces pathological damage in the cerebral cortex, enhances neuroprotective functions, and activates the BDNF/TrkB pathway (Liu X. et al., 2023). This is consistent with the findings of Ni et al., who reported that AS-IV promotes neurogenesis in MCAO rats by upregulating the expression of the BNDF/TrkB signaling pathway (Ni et al., 2020). Another study demonstrated that AS-IV mediates the inhibition of radiation-induced morphological damage and cognitive dysfunction in mouse neurons by activating the BDNF-TrkB pathway, thereby exerting a neuroprotective effect (Liu X. et al., 2023).

### AMPK/mTOR

After stroke, cerebral ischemia and hypoxia lead to energy metabolism disorder. AMPK/mTOR cooperates with autophagy to fine-tune metabolic activities and exert anti-apoptotic and anti-inflammatory effects in response to stress (Chun and Kim, 2021; Sun Z. et al., 2020). mTORC1 and mTORC2 are two distinct mTOR complexes (Szwed et al., 2021). Under hypoxic-ischemic conditions, activated AMPK can inhibit mTORC1 and then regulate the downstream substrates (Nacarkucuk et al., 2024). Following cerebral ischemia, AS-IV improves OGD/R injury by inhibiting AMPK/mTOR-triggered autophagy and mitochondrial-mediated apoptosis (Hao et al., 2023). In addition, AS-IV can induce AMPK activation, simultaneously reduce the levels of phosphorylated mTOR and hypoxia-inducible factor-1alpha, and promote the tissue remodeling after IS through the polarization of microglia in a dependent metabolic pathway (Li MC. et al., 2024). In another study, it was found that AS-IV can effectively activate epidermal growth factor receptor/MAPK signaling pathway, promote the proliferation and neurogenesis of NSCs in rats with transient cerebral ischemia, and improve the repair of neurological function in rats with IS (Chen et al., 2019).

### Others

AS-IV exerts neuroprotective effects against stroke through multi-target and multi-pathway mechanisms. Beyond the classical pathways described above, it also exerts neuroprotective effects via additional pathways. It can activate the TGF-β/small mother against decapentaplegic (Smad) signaling pathway, improving cognitive function after cerebral infarction by regulating the levels of TGF-β, Smad1, Smad3, and Smad7 (Li L. et al., 2020). AS-IV inhibits the upregulation of matrix metalloproteinase-9 and aquaporin-4 to reduce brain edema, Li et al. (2013) and improves neurological deficits and reduces infarct volume via the SIRT1/MAPT pathway (Shi et al., 2021). It also regulates the hypothalamic-pituitary-adrenal axis to ameliorate peripheral immune suppression (Zou et al., 2022) and reverses intestinal microbiota disorders to alleviate autophagy and OS (Xu et al., 2018). AS-IV dual-inhibits the death receptor pathway (Fas, FasL, Caspase-8) and mitochondrial apoptosis pathway (Bax/Bcl-2, Caspase-3, etc.), Yin et al. (2020) and activates the PKA/CREB and Janus tyrosine kinase 2/STAT 3 signaling pathways to enhance neuronal survival and antioxidant capacity (Xue et al., 2019; Xu et al., 2020). In terms of brain remodeling and repair, AS-IV may downregulate IL-17 expression via the Wnt pathway (Sun et al., 2020b). AS-IV promotes the activation of STAT3 by upregulating the expression of fat mass and obesity-associated protein, which reduces the N6-methyladenosine modification level of Acyl-CoA synthetase long-chain family member 4, thereby improving IS-induced neuronal damage by inhibiting ferroptosis (Jin et al., 2023).

In addition, proteomic analysis revealed that AS-IV may play a neuroprotective role by regulating the expression of Aldolase C, Dihydrolipoamide dehydrogenase and Triose-phosphate isomerase (Lo et al., 2021). Wei et al. (2023) elucidated the mechanism of AS-IV in treating ischemic brain injury by integrating transcriptomics, proteomics, and metabolomics strategies. They identified key metabolites including 3,4-dihydroxy-L-phenylalanine, 2-aminomuconic semialdehyde, and (R)-3-hydroxybutyrate, and affected pathways including tyrosine metabolism, tryptophan metabolism, butyrate metabolism, and purine metabolism. Core targets include adenine phosphoribosyltransferase, AICAR transformylase/IMP cyclohydrolase, acid alpha-glucosidase, galactokinase, beta-galactosidase, malic enzyme 2, and hexosaminidase A.

These mechanisms collectively form a comprehensive neuroprotective network of AS-IV in anti-inflammation, anti-apoptosis, repair promotion, and maintenance of neurovascular unit integrity.

## Effects of AS-IV combined with other natural plant metabolites on neuroprotection

The combined application of AS-IV with other natural plant metabolites has shown significant synergistic effects in neuroprotection against stroke. As a classic combination, Astragalus membranaceus and Carthamus tinctorius L. (Asteraceae; the dried florets of Carthamus tinctorius) are widely used in the treatment of cardiovascular and cerebrovascular diseases, characterized by multi-metabolite and multi-target properties (Wang K. et al., 2022). A study explored the intervention effects of the Huangqi-Honghua combination (comprising Astragalus membranaceus and Carthamus tinctorius) and its main metabolites (AS-IV and hydroxysafflor yellow A, HSYA) in a rat model of CIRI with qi deficiency and blood stasis. The results showed that the combination of AS-IV and HSYA significantly reduced whole blood viscosity and plasma viscosity in rats, improved neurological deficits, and reduced infarct volume. Simultaneously, by upregulating Nrf2 expression, enhancing the activity of antioxidant enzymes (e.g., superoxide dismutase), and reducing the production of malondialdehyde and ROS, the combination exerted neuroprotective effects, and confirmed the traditional efficacy of Huangqi-Honghua in the treatment of stroke with qi deficiency and blood stasis (Cao et al., 2014). Another study on the MCAO model systematically investigated the intervention effects and mechanisms of AS-IV and HSYA alone and in combination. The results showed that the combined treatment was significantly more effective than single-drug intervention. Combined medication can significantly reduce the cerebral infarction volume from (44 ± 5) % to (24 ± 2) %, the neurological function scores from (2.83 ± 0.52) to (1.42 ± 0.49), and the brain water content from (83.67 ± 2.34) % to (57.33 ± 2.58) %. Additionally, the combination more effectively inhibited the NF-κB/NLRP3/Caspase-1/GSDMD signaling pathway, thereby reducing cell death (Hou et al., 2024).

A further study systematically explored the pharmacokinetic characteristics of eight active metabolites of Huangqi-Honghua (including AS-IV, HSYA, and calycoside.) in the treatment of CIRI and their regulatory effects on neurotransmitters using blood-brain dual-channel microdialysis combined with liquid chromatography-tandem mass spectrometry. The results showed that all active metabolites could cross the BBB of CIRI rats and significantly regulate the release of five neurotransmitters (e.g., glutamic acid, γ-aminobutyric acid, and dopamine). The results showed that all active metabolites could cross the BBB of CIRI rats and significantly regulate the release of five neurotransmitters (e.g., glutamic acid, γ-aminobutyric acid, and dopamine). Among these, AS-IV and HSYA exhibited superior regulatory effects, with glutamic acid identified as the primary neurotransmitter target, which proves that Huangqi-Honghua plays an anti-CIRI effects by improving neurotransmitter imbalance (Du et al., 2022). The study also found a lag between the peak plasma concentration of Huangqi-Honghua and its maximum therapeutic effect, and noted that the effective concentration range of some metabolites (e.g., AS-IV and calycoside) in blood and brain microdialysis is narrow, which highlighted the necessity of careful dose control in clinical application (Du et al., 2022).

Huang et al. (2015) conducted a study to elucidate the protective efficacy and related mechanisms of the combination of AS-IV and major active metabolites of Panax notoginseng (Burk.) F.H.Chen. (Araliaceae; the dried root and rhizome of Panax notoginseng), namely ginsenosides Rg1, Rb1 and notoginsenoside R1, in a mice model of CIRI. The results showed that the combination significantly enhanced protective effects by inhibiting the activation of the NF-κB and tyrosine kinase 1/signal transducer and activator of transcription-1 signaling pathways, regulating endoplasmic reticulum stress (regulating glucose regulated protein 78, caspase-12 and other proteins), and exerting anti-inflammatory (inhibiting TNF-α, IL-1β, etc.) and anti-apoptotic effects (improving neuronal survival rate and reducing apoptosis rate), with superior synergistic effects. A study by Tang B. et al. (2021) confirmed using the MCAO model that the neuroprotective effect of AS- IV combined with Panax notoginseng saponins is significantly better than that of single-drug treatment. The combination significantly reduced neurological deficit scores, infarct volume, and cortical cell injury rate, improved cortical pathological damage, and more effectively reduced the levels of proteins associated with pyroptosis and necroptosis. Its neuroprotective effect is closely related to the inhibition of these two forms of cell death modes.

Furthermore, a study explored the protective mechanism of AS- IV combined with ligustrazine of Ligusticum chuanxiong Hort. (Apiaceae; the dried rhizome of Ligusticum chuanxiong) against CIRI (Chen X. et al., 2024). The results showed that 7 days of combined intervention in a rat CIRI model significantly improved neurological and cognitive functions, restored cerebral blood flow, reduced infarct volume, and alleviated cortical neuronal and mitochondrial damage. In an OGD/R model of SH-SY5Y cells, the combination enhanced cell viability, reduced lactate dehydrogenase and ROS release, and increased adenosine triphosphate content and mitochondrial membrane potential. Its core mechanism involves regulating the SUMOylation process associated with mitochondrial dynamics. By reducing the SUMO-1 modification of dynamin-related protein 1(Drp1), increasing SUMO-2/3 modification of Drp1, downregulating mitochondrial fission proteins (e.g., Drp1 and fission 1 protein), upregulating mitochondrial fusion proteins (e.g., OPA1 and mitofusin-1), and regulating the expression of sentrin-specific protease family proteins, the combination improves mitochondrial homeostasis and exerts anti-CIRI effects.

These findings suggest that the combination of AS-IV with other natural plant metabolites provides novel strategies for multi-target stroke treatment through integrated mechanisms (mitochondrial protection, anti-inflammation, antioxidation, neurotransmitter regulation, and metabolic control), demonstrating significant potential for clinical translation.

## Research hotspots and trend topics

**FIGURE 2:**
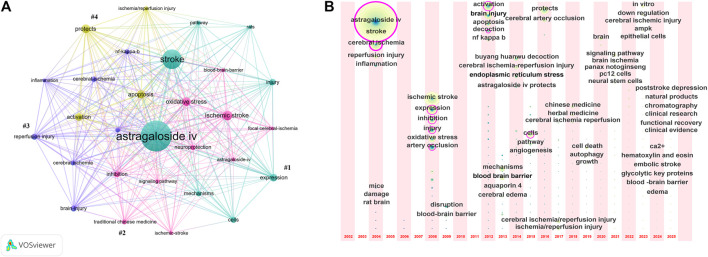
**(A)** Co-occurrence diagram of research keywords of AS-IV applied to stroke. **(B)** Keywords time zone map of AS-IV applied to stroke.

Keyword co-occurrence analysis can reveal meaningful knowledge metabolites and perspectives based on the pattern and intensity of links between keywords, and identify hotspots and latest trends in the research field by revealing knowledge mapping (Yu X. et al., 2025). In the current research, a total of 629 keywords were extracted by VOSviewer software, among which 29 keywords that appeared at least 7 times were selected for visual analysis, with each color representing a category, resulting in four clusters (Figure 2A). As shown in the figure, research in this field primarily focuses on the application of AS-IV in IS, particularly in CIRI models, with the core research goal of clarifying its neuroprotective mechanisms at the molecular level. Inhibiting apoptosis, reducing OS and controlling neuroinflammation are the primary current research hotspots.

Figure 2B dynamically reflects the evolution of research hotspots in this field over time. As shown, research on AS-IV in stroke has undergone four evolutionary stages: basic pathological anchoring, deepening of molecular mechanism, interdisciplinary expansion and clinical transformation orientation, with different focuses at each stage. From 2002 to 2007, the preliminary verification of drug-disease relationship was completed with the core pathological injuries of stroke (e.g., ischemia-reperfusion injury and inflammation) and animal model verification. During the period of 2008–2012, in-depth breakthroughs in cellular/molecular mechanisms (e.g., OS, apoptosis, and NF-κB). In 2013–2018, further analysis of subclinical pathological targets (e.g., endoplasmic reticulum stress, BBB, autophagy and brain edema), integration of multidisciplinary methods to overcome the limitations of single mechanism explanation, and incorporation of traditional Chinese medicine characteristics (e.g., decoctions and Buyang Huanwu Decoction) to consolidate the modern pharmacological research paradigm guided by traditional Chinese medicine theory. Since 2019, the research in this field has turned to the two-way exploration of clinical transformation prospects and frontier mechanism innovation. While no clinical trials of AS-IV alone in stroke patients have been identified, the emergence of keywords such as “clinical research,” “clinical evidence,” “post-stroke depression,” and “functional recovery” reflects the future direction of transitioning from basic mechanisms to clinical efficacy verification and improving long-term patient prognosis, aligning with clinical needs for comprehensive stroke management and frontier trends in life sciences. Overall, this reflects a typical research trend of transforming natural botanical drug monomers into modern medicines.

## Challenges and prospects

At present, AS-IV has not been used as an independent drug in clinical practice. Although extensive preclinical studies have demonstrated its potential for multi-target regulation in stroke, suggesting it may serve as an effective natural product for stroke prevention and treatment by inhibiting neuroinflammation and immune imbalance, regulating OS and ferroptosis, inhibiting neuronal apoptosis, regulating autophagy and media-melting function, promoting neurogenesis and synaptic plasticity, promoting angiogenesis and repairing BBB. However, research on AS-IV in stroke prevention and treatment remains limited, and several challenges persist.

First, the neuroprotective effects of AS-IV involve the regulation of multiple pathological links and signal pathways, with its biological effects primarily mediated by two interconnected yet independent mechanisms: immunomodulation and direct binding to cellular targets. The former focuses on regulating the function of immune cells (e.g., macrophages, microglia, and T lymphocytes) and cytokine secretion. The latter involves direct binding to potential protein targets (e.g., TNF-α, VEGF receptor-2, and phosphatidylinositol 3-kinase γ). The sugar chain of AS-IV can act as a hydrogen bond donor to form stable interactions with residues in the active site of TNF-α, while the aglycone moiety can insert into the hydrophobic pocket of VEGF receptor-2 to inhibit its signal activation. Although no direct structural biology evidence is currently available, preliminary molecular docking simulations suggest that the C-3 and C-20 hydroxyl groups of AS-IV may form hydrogen bonds with the active site of phosphatidylinositol 3-kinase γ, requiring verification via X-ray crystallography or cryo-electron microscopy in future studies.

However, existing research has not clarified the interaction mechanisms between core targets and pathways, the upstream-downstream regulatory relationships of each pathway, the dominant mechanisms in different pathological stages, or the mechanisms underlying immune cell metabolic reprogramming. This ambiguity in core targets has limited its translational value. For example, in the regulation of neuroinflammation, AS-IV promotes the polarization of microglia from M1 to M2, but the association between energy metabolism reprogramming and immune cell metabolic phenotypes in this process remains unclear, preventing accurate localization of intervention targets and limiting the clinical translational value of its mechanisms. A comprehensive understanding of the potential mechanisms and targets of AS-IV in stroke prevention and treatment is critical for developing effective therapeutic strategies. Future research should construct a regulatory network of targets, pathways, and pathological processes using multi-omics technologies (e.g., transcriptomics and proteomics) combined with single-cell sequencing and bioinformatics analysis to clarify the specific effects of AS-IV on different brain cell types, accurately identify core regulatory nodes, and provide mechanistic support for optimizing intervention strategies.

Secondly, significant gaps exist in pharmacokinetic studies of AS-IV, its absorption, distribution, metabolism, and excretion characteristics have not been systematically elucidated, particularly its low brain bioavailability, unclear effective therapeutic concentration, and insufficient exploration of toxicity (including acute toxicity, chronic toxicity, and organ-specific toxicity). These factors have become key obstacles limiting its therapeutic efficacy. From a physicochemical perspective, AS-IV exhibits good thermal stability but poor water solubility, resulting in low gastrointestinal absorption efficiency after oral administration. More crucially, its BBB penetration ability is weak (0.49 ± 0.03), making it difficult to reach effective therapeutic concentrations in ischemic brain regions. Additionally, no studies have investigated whether AS-IV accumulates in non-target organs (e.g., liver and kidney) due to poor targeting, and the safety window between therapeutic and toxic doses remains unclear. Furthermore, no comparative studies on the pharmacokinetics and toxicity of AS-IV across different stroke subtypes, animal models, or administration routes have been conducted. For example, it remains unknown whether intravenous injection increases the risk of nephrotoxicity compared to oral administration, or whether long-term administration in chronic stroke models induces immune-related side effects. These uncertainties prevent the determination of optimal dosage regimens and accurate dose-effect relationships, and hinder the evaluation of long-term application safety, seriously affecting the scientificity and reliability of efficacy evaluations and the clinical translation process.

To address these bottlenecks, the development of novel nano-drug delivery systems is necessary. By combining carriers (e.g., liposomes and polymer nanoparticles) with BBB-specific receptor ligand modification, targeted delivery of AS-IV to the brain can be achieved, reducing drug distribution in non-target organs and thereby lowering the risk of off-target toxicity. Simultaneously, the use of pH-sensitive or redox-responsive carriers can enable site-specific drug release in ischemic regions, improving local effective concentrations and avoiding excessive accumulation in normal brain tissue. In addition, leveraging blood-brain-like barrier chip models and *in vitro* toxicity evaluation models (e.g., liver and kidney cell models) can facilitate the simultaneous screening and optimization of drug delivery strategies and *in vitro* toxicity evaluation, addressing the core issues of low AS-IV bioavailability and unclear toxicity risks (Lall et al., 2025; Kistemaker et al., 2025).

Finally, the existing research is mostly limited to *in vitro* cell experiments and animal models, lacking high-quality clinical research evidence, resulting in multi-dimensional bottlenecks for clinical translation. Significant differences exist between preclinical models and human stroke pathology: animal models fail to fully simulate the complex pathophysiological processes of human stroke (e.g., comorbidities and individual genetic differences), making it difficult to replicate the neuroprotective effects observed in animal experiments in humans. AS-IV lacks a standardized quality control system: differences in extraction and purification processes lead to fluctuations in purity and impurity content, affecting batch-to-batch efficacy stability and increasing the risk of clinical application. Insufficient long-term safety and efficacy data: existing research focuses on short-term intervention effects, with no evaluation of the potential impacts of long-term AS-IV administration on liver and kidney function or immunosuppression risk. At the same time, lack of individualized treatment protocols, and the optimal dose and course of treatment for patients with different ages, genders and stroke severities remain unclear, preventing precision treatment. In addition, the synergistic effect and potential interaction between AS-IV and other drugs (e.g., thrombolytics and antiplatelet agents) have not been verified, which further limits its clinical application scenarios.

To address these issues, a full-chain standardization system should be established, clarify the authentic producing areas of medicinal materials, optimize the extraction processes, and ensure AS-IV purity and batch consistency using methods such as HPLC-MS/MS. In addition, quasi-clinical animal models (e.g., aged mice with chronic inflammation and patient-derived xenotransplantation models) to verify the *in vivo* efficacy and safety of AS-IV, bridging the gap between *in vitro* research and clinical application. Design multi-center, randomized, double-blind controlled clinical trials focusing on IS patients to evaluate its efficacy in improving neurological deficits and long-term safety. At the same time, combine patient clinical characteristics and molecular markers to develop an efficacy prediction model using artificial intelligence, formulate individualized medication plans, and explore combined applications with intravenous thrombolysis and mechanical embolectomy, promoting the transformation of AS-IV from broad-spectrum intervention to precision treatment.

## Conclusion

AS-IV exerts neuroprotective effects by inhibiting neuroinflammation and immune imbalance, regulating OS and ferroptosis, suppressing neuronal apoptosis, modulating autophagy and lysosomal function, promoting neurogenesis and synaptic plasticity, facilitating angiogenesis, and repairing the BBB, acting through multiple signaling pathways. The combination of AS-IV with HSYA, ligustrazine, and the main active metabolites of Panax notoginseng exhibits synergistic effects, significantly enhancing therapeutic efficacy. Notably, despite current challenges, the pleiotropy of AS-IV provides new insights for stroke treatment, and breakthroughs are needed in mechanism deepening, pharmacokinetic optimization, clinical verification and standardization construction. In summary, as an accessible and low-cost natural neuroprotectant, AS-IV holds significant development value in this field and serves as a potential candidate drug for adjuvant stroke therapy, warranting further exploration in future studies.
